# Supplementary material for: A potato late blight pathogen effector interacts with ENTH-domain protein TOL9a and an activated helper NLR to suppress immunity
Source: Sci Adv. 2026 Jun 5;12(23):eaea4500. doi: 10.1126/sciadv.aea4500 (PMC13240173; doi:10.1126/sciadv.aea4500)
Supplement: Supplementary file 1 — Figs. S1 to S9 Table S1 Legends for tables S2 to S4 Legends for movies S1 and S2 Legends for data S1 to S4 References [file sciadv.aea4500_sm.pdf]

Science Advances
AAAS

Supplementary Materials for

A potato late blight pathogen effector interacts with ENTH-domain protein TOL9a and an activated helper NLR to suppress immunity

Jogi Madhuprakash *et al.*

Corresponding author: Mauricio P. Contreras, mauricio.contreras@zmbp.uni-tuebingen.de;
Sophien Kamoun, sophien.kamoun@tsl.ac.uk

Sci. Adv. **12**, eaea4500 (2026)
DOI: 10.1126/sciadv.aea4500

The PDF file includes:

Figs. S1 to S9
Table S1
Legends for tables S2 to S4
Legends for movies S1 and S2
Legends for data S1 to S4
References

Other Supplementary Material for this manuscript includes the following:

Tables S2 to S4
Movies S1 and S2
Data S1 to S4

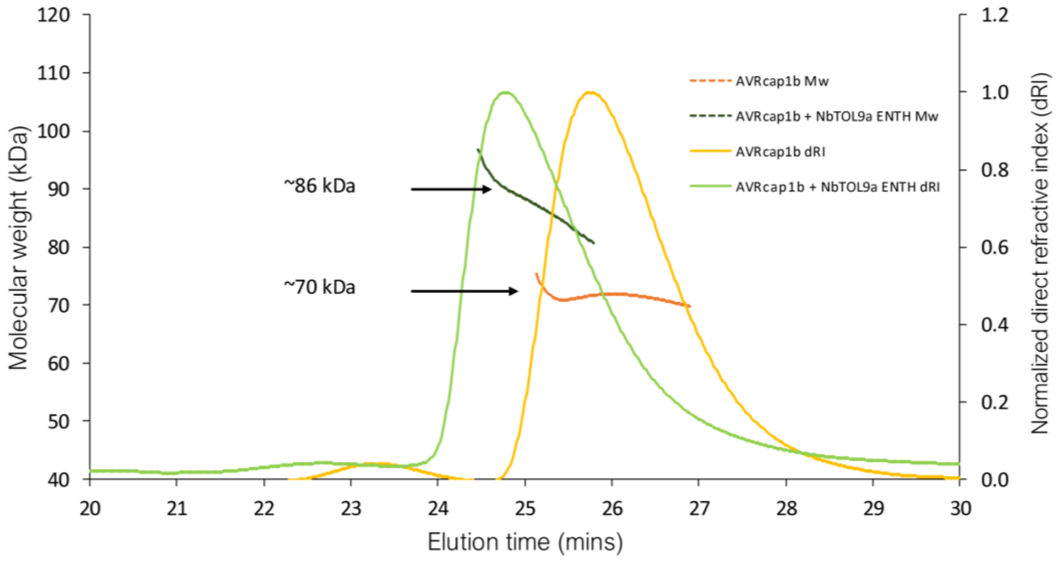

Fig. S1. SEC-MALS confirms complex formation between AVRcap1b and NbTOL9a^{ENTH}. Size-exclusion chromatography coupled to multi-angle light scattering (SEC-MALS) was used to determine the molar mass of AVRcap1b alone (yellow) and in complex with NbTOL9a^{ENTH} (green). Normalized differential refractive index (dRI) traces represent elution profiles from a Superdex 200 Increase 10/300 GL column. Dashed lines indicate the calculated molecular weight (kDa) across the elution peaks. Incubation of AVRcap1b with NbTOL9a^{ENTH} resulted in a shift in elution volume and an increase in calculated molecular weight from ~70 kDa (AVRcap1b alone) to ~86 kDa, consistent with the formation of a 1:1 complex in solution.

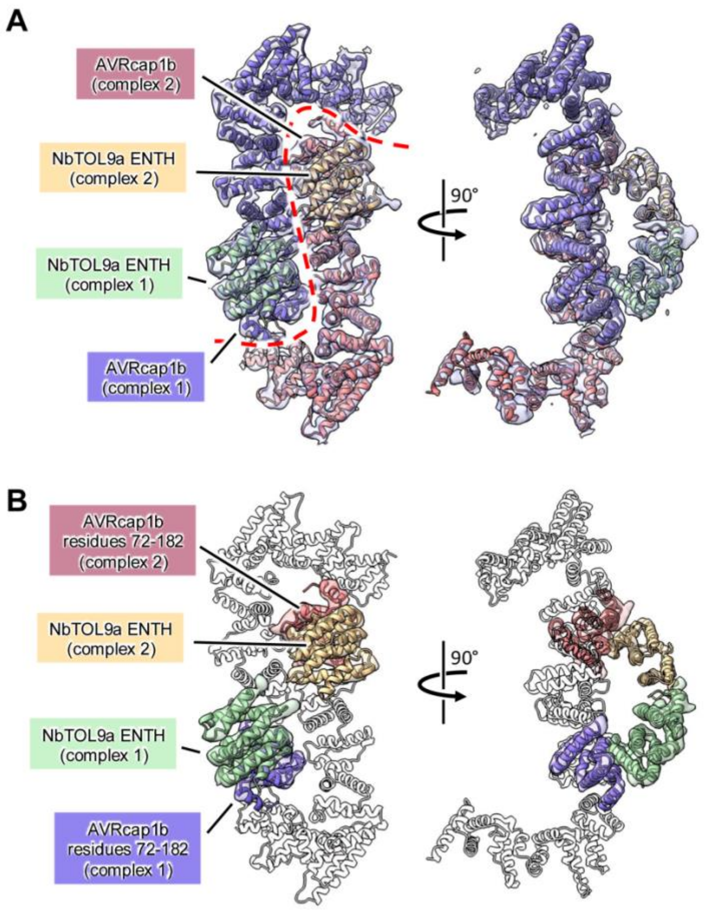

Fig. S2: Crystallographic evidence supporting the structural model of the AVRcap1b – NbTOL9a^{ENTH} complex.

The crystallographic asymmetric unit contains two copies of the AVRcap1b – NbTOL9a^{ENTH} complex that are related by two-fold non-crystallographic symmetry (shown as orthogonal views). The left-hand images for both panels show the view looking down this two-fold axis, whilst the axis runs horizontally in the right-hand images; the red dashed line in the top left image demarcates the boundary between the two complexes. (**A**) Fit of the refined model, colour-coded by subunit, to the final $2m\mathrm{F}_{obs}$-$D\mathrm{F}_{calc}$ electron density map calculated at 4.1 Å resolution and contoured at ~1.5σ (semi-transparent pale blue surface). (**B**) A series of omit difference maps ($m\mathrm{F}_{obs}$-$D\mathrm{F}_{calc}$) were calculated by separately removing parts of the final structure and re-refining to convergence. The omitted parts were the N-terminal portion of each copy of AVRcap1b (residues 72-182) and each of the full NbTOL9a^{ENTH} domains, yielding four separate maps. These are displayed together and coloured according to the part of the structure that was omitted. These are all calculated at 4.1 Å resolution and contoured at ~2.0σ. The remainder of the structure is white and transparent. See Supplementary **Movies S1** and **S2** for rotating views of the electron density and omit difference maps shown in panels A and B, respectively.

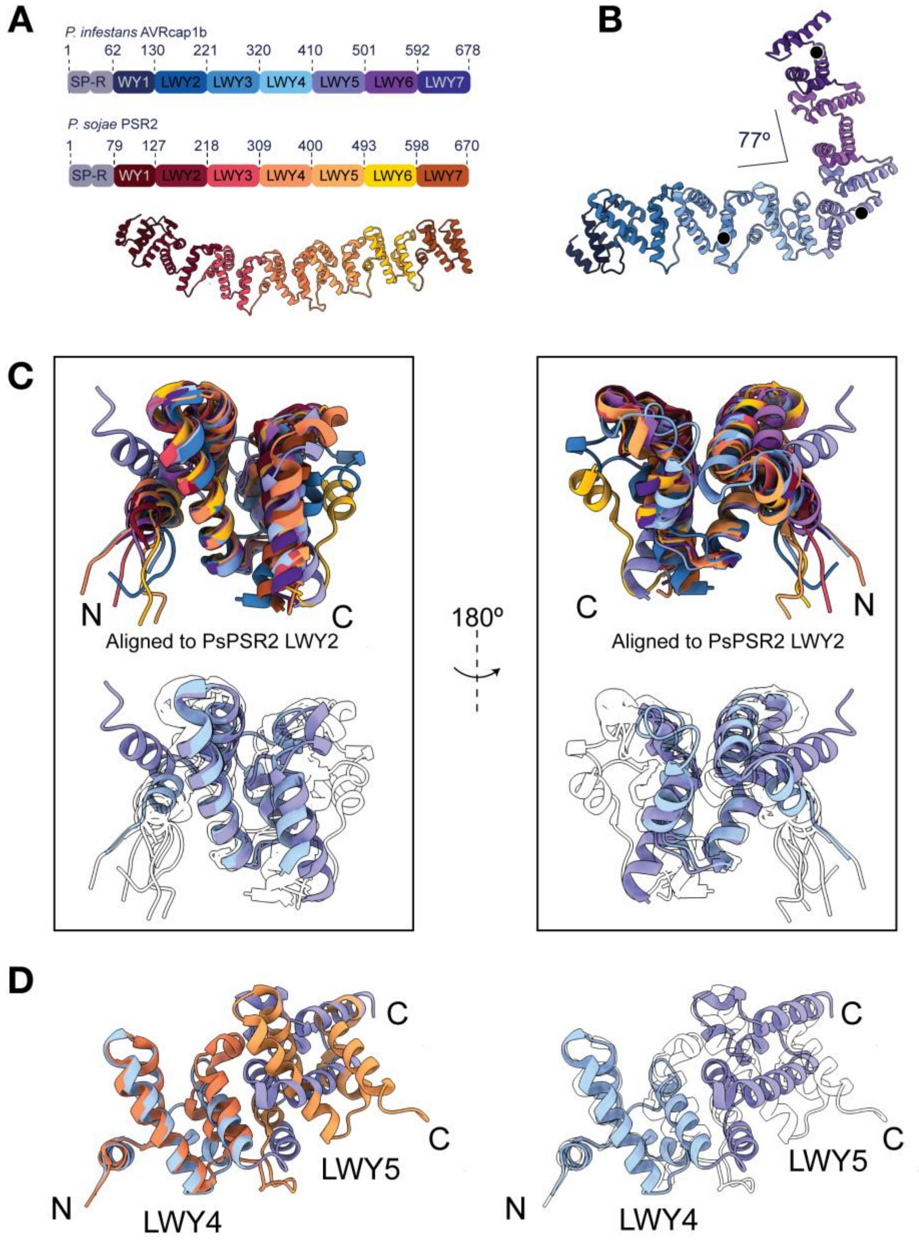

Fig. S3: LWY4 and LWY5 of AVRcap1b are structurally distinct from other LWY modules. (**A**) Schematic representation of *P. infestans* AVRcap1b and *P. sojae* PSR2. Individual structural repeat units of each effector are color-coded as indicated in the schematic, which also denotes the amino acid boundaries for each repeat/domain. The schematic highlights the signal peptide (SP) and RXLR-DEER motif (R) at the N-terminus. Overall structure of *P. sojae* PSR2 is also included (PDB ID: 5GNC). (**B**) Overall L-shaped structure of AVRcap1b, highlighting the 77º angle introduced by LWY4 and LWY5 repeats. Black dots indicate amino acids selected to calculate the angle. (**C**) Two different views of a structural alignment of all LWY repeats from PSR2 and AVRcap1b. Bottom images feature the same alignment with all repeats made transparent and LWY4 and LWY5 from AVRcap1b in color. (**D**) Structural alignment of LWY4 and LWY5 of PSR2 and AVRcap1b. Color coding is as indicated in the schematics in panel (**A**).

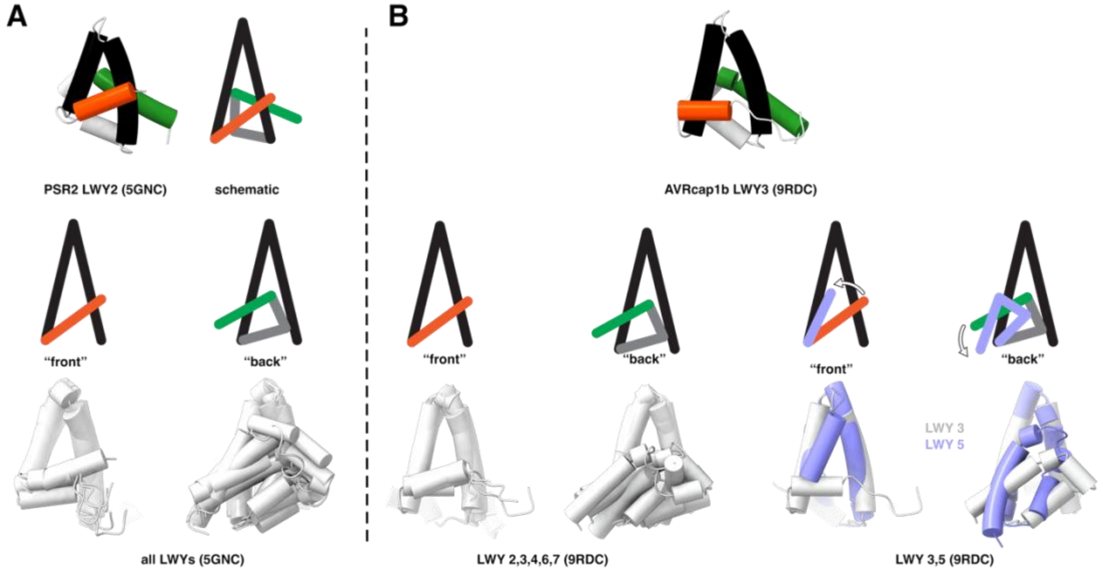

Fig. S4: Structural analysis of LWY repeats reveals distinct structural conformation of *P. infestans* LWY5.

(A) Top: Overall structural arrangement of alpha-helices in *Phytophthora sojae* PSR2 (PDB: 5GNC) LWY2 repeat with α1 helix in red and α5 helix in green. Bottom: Structural alignment of all LWYs of *P. sojae* PSR2 LWY repeats including cartoon representation of the overall arrangement of alpha-helices. (B) Top: Overall structural arrangement of alpha-helices in *Phytophthora infestans* AVRcap1b LWY3 repeat. Bottom left: Structural alignment of *Phytophthora infestans* AVRcap1b LWY domains 2-4 and 6-7, including cartoon representation of the overall arrangement of alpha-helices. Bottom right: Structural alignment of *P. infestans* AVRcap1b LWY repeats 3 and 5. LWY4 is colored in gray, whereas LWY5 is colored in purple. Cartoon representation highlights key rotations seen in α1 and α5 helices which are responsible for the bend in AVRcap1b.

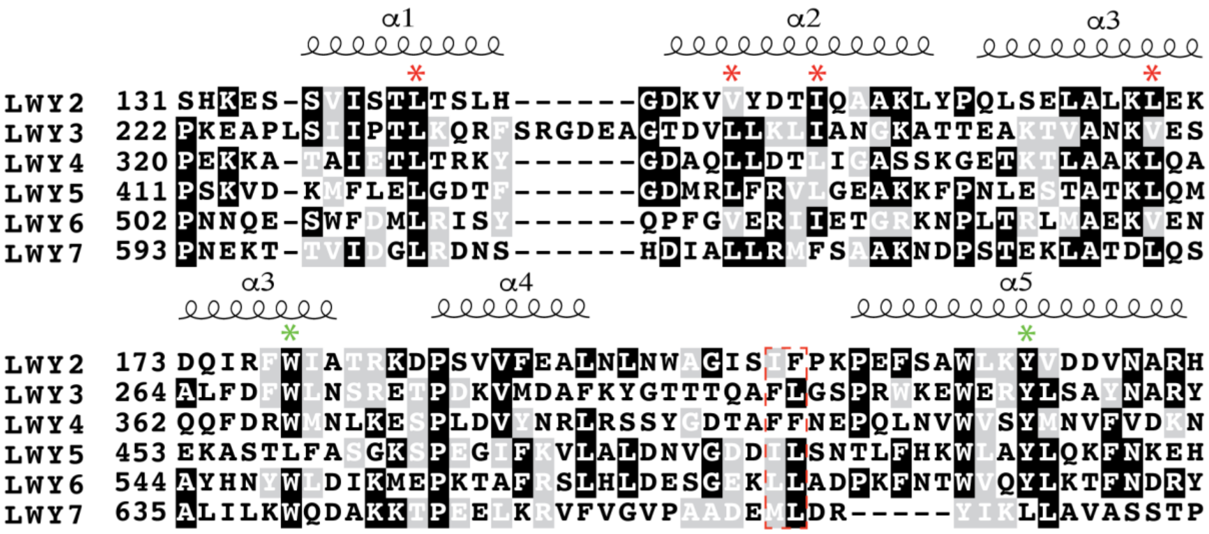

Fig. S5: Amino acid sequence alignment of all LWY modules of AVRcap1b

The conserved hydrophobic Loop$^{4-5}$ is highlighted with a red dashed line. The hydrophobic residues that make up the conserved hydrophobic pocket in each LWY module are shown with red asterisks. Green asterisks highlight the conserved W and Y residues. Secondary structure is shown at the top.

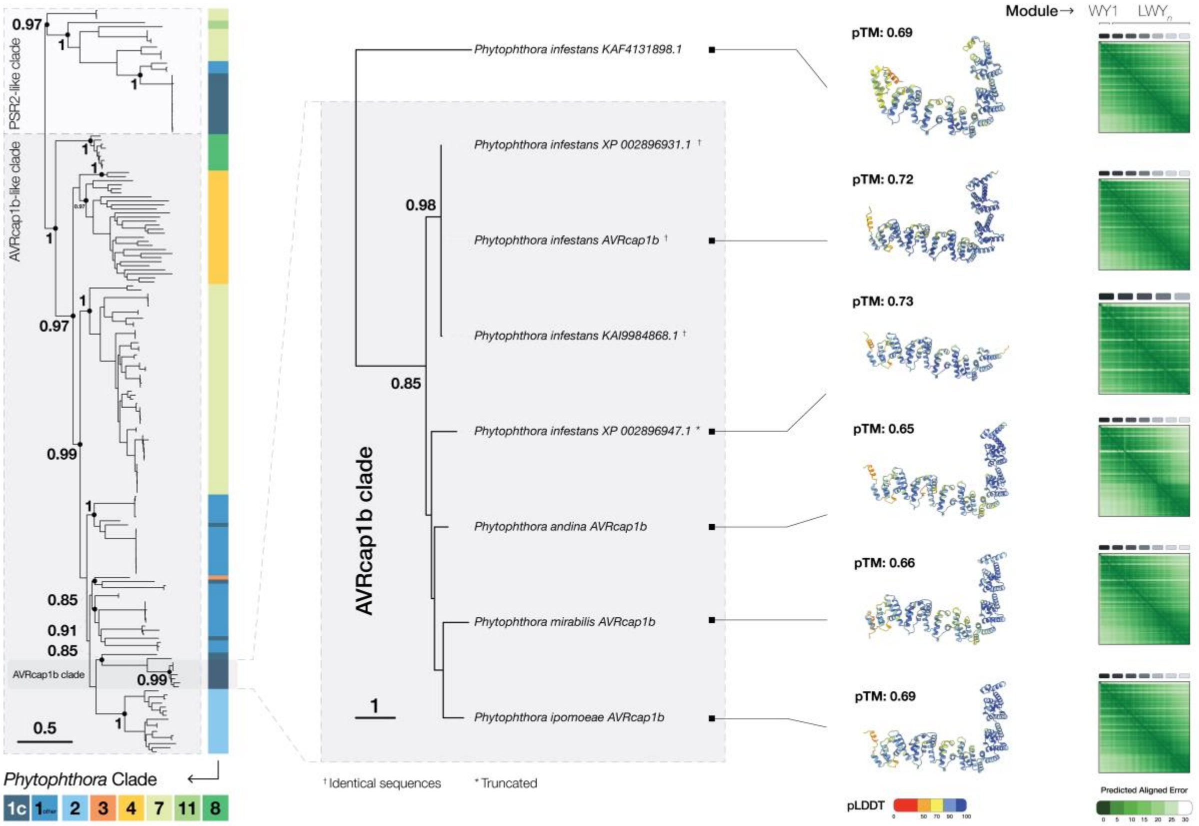

Fig. S6. Clade 1c AVRcap1b orthologs all share the same L-shaped effector fold.
A magnified view of the AVRcap1b subclade from the phylogenetic tree of AVRcap1b-like and PSR2-like sequences shows that all clade 1c orthologs adopt the characteristic L-shaped fold, consistent with other AVRcap1b-like effectors. One exception is the *P. infestans* sequence XP_002896947.1, which was predicted to have a shorter, stick-like structure due to the absence of LWY6 and LWY7 repeats. Structural modeling was performed using AlphaFold 3 (*47*).

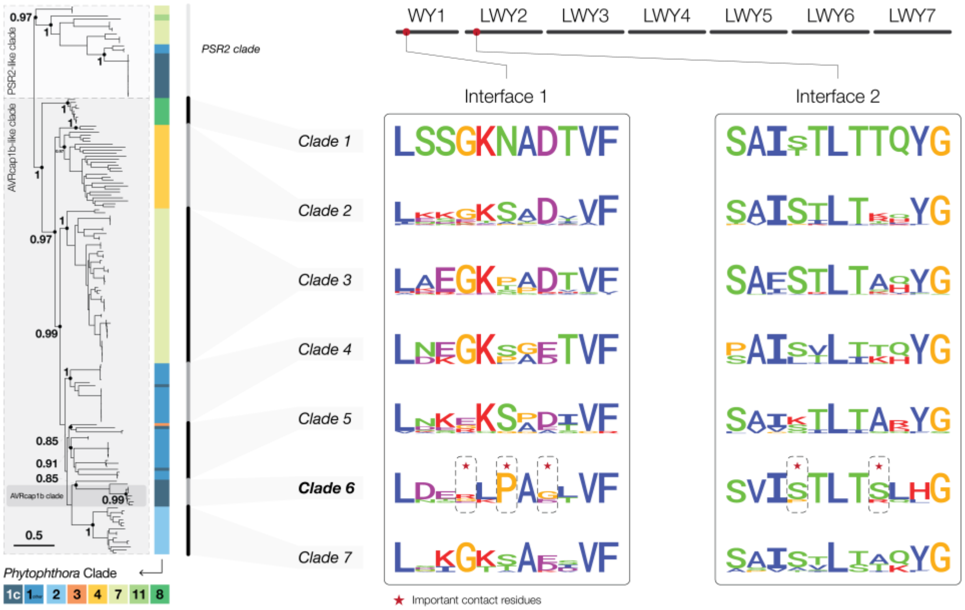

Fig. S7. NbTOL9a interaction interfaces show variability across AVRcap1b clades.
The AVRcap1b-like clade was divided into seven subclades based on well-supported phylogenetic branches and Phytophthora clade annotations. Sequence logo plots were generated for two NbTOL9a ENTH domain binding interfaces on AVRcap1b, located on the WY1 and LWY2 repeats, respectively, across all subclades. Both interfaces exhibited variability within and between subclades. In clade 6, which includes AVRcap1b and its clade 1c orthologs, only one of the five key contact residues—corresponding to P92 (interface 1)—was conserved across all sequences.

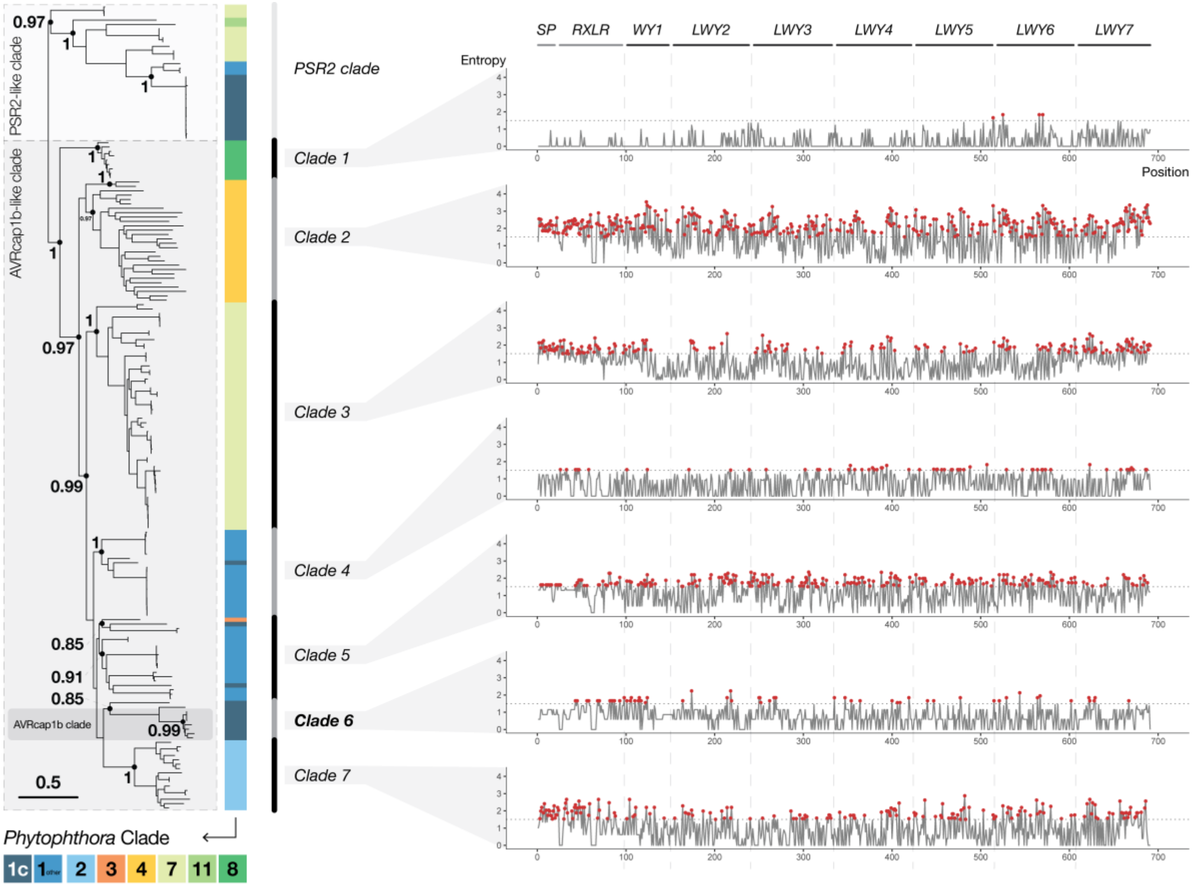

Fig. S8. AVRcap1b subclades exhibit variable Shannon's entropy patterns.

Entropy was calculated across AVRcap1b-like sequences grouped into subclades. Sites with entropy values >1.5 were considered highly variable. WY and LWY modules exhibited extensive variation both within and between clades. In clade 6, which includes AVRcap1b and its clade 1c orthologs, WY1 was the most variable module, with 10 highly variable sites compared to other LWY modules (**Table S2**).

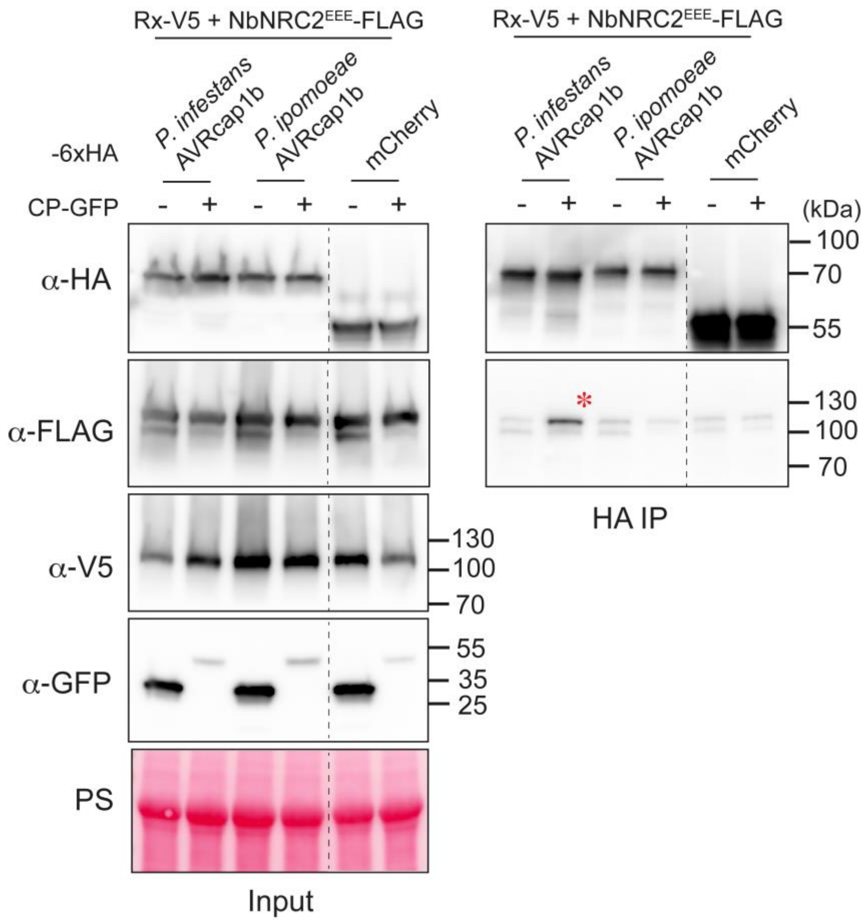

Fig. S9: Unlike *P. infestans* AVRcap1b, the *P. ipomoeae* ortholog does not associate with Rx/CP-activated NbNRC2.

CoIP assays between resting state or activated NbNRC2 and AVRcap1b variants. C-terminally 3xFLAG-tagged NbNRC2 was coexpressed with C-terminally V5-tagged Rx and C-terminally 6xHA-tagged AVRcap1b. the Rx/NbNRC2 system was activated with C-terminally GFP tagged PVX CP. Free GFP used as a negative control for activation. IPs were performed with agarose beads conjugated to HA antibodies (HA IP). Total protein extracts were immunoblotted with the antisera labeled on the left. Approximate molecular weights (in kilodalton) of the proteins are shown on the right. Red asterisk indicates AVRcap1b association with Rx/CP activated NbNRC2^{EEE}. Rubisco loading control was carried out using Ponceau stain (PS). The experiment was repeated three times with similar results.

Table S1: Summary of X-ray data and model parameters for AVRcap1b – NbTOL9a^{ENTH} complex.

**Data collection**	
Diamond Light Source beamline	I04
Wavelength (Å)	0.9795
Detector	Eiger2 XE 16M
Resolution range (Å)	79.57 – 4.10 (4.49 – 4.10)
Space group	$P2_12_12_1$
Cell parameters (Å)	a = 85.9, b = 136.9, c = 195.6
Total no. of measured intensities	242580 (59716)
Unique reflections	18772 (4401)
Multiplicity	12.9 (13.6)
Mean $I/\sigma(I)$	9.6 (1.0)
Completeness (%)	100.0 (100.0)
R_{merge} [a]	0.106 (2.126)
R_{meas} [b]	0.110 (2.208)
$CC_{½}$ [c]	1.000 (0.518)
**Refinement**	
Resolution range (Å)	79.57 – 4.10 (4.21 – 4.10)
Reflections: working/free[d]	17765/938
R_{work}/ R_{free} [e]	0.248/0.286 (0.419/0.431)
MolProbity score/Clashscore[f]	1.39/0.97
Ramachandran plot: favoured/allowed/disallowed[f] (%)	98.2/1.8/0.0
R.m.s. bond distance deviation (Å)	0.005
R.m.s. bond angle deviation (°)	1.53
AVRcap1b – chains/residue ranges	A,B/78-675,78-675
ENTH – chains/residue ranges	C,D/1-138,3-138
PDB accession code	9RDC

Values in parentheses are for the outer resolution shell.

[a] $R_{merge} = \sum_{hkl} \sum_i |I_i(hkl) - \langle I(hkl)\rangle| / \sum_{hkl} \sum_i I_i(hkl)$.

[b] $R_{meas} = \sum_{hkl} [N/(N-1)]^{1/2} \times \sum_i |I_i(hkl) - \langle I(hkl)\rangle| / \sum_{hkl} \sum_i I_i(hkl)$, where $I_i(hkl)$ is the ith observation of reflection hkl, $\langle I(hkl)\rangle$ is the weighted average intensity for all observations i of reflection hkl and N is the number of observations of reflection hkl.

[c] $CC_{½}$ is the correlation coefficient between symmetry equivalent intensities from random halves of the dataset.

[d] The data set was split into "working" and "free" sets consisting of 95 and 5% of the data respectively. The free set was not used for refinement.

[e] The R-factors R_{work} and R_{free} are calculated as follows: $R = \sum(|F_{obs} - F_{calc}|)/\sum|F_{obs}|$, where F_{obs} and F_{calc} are the observed and calculated structure factor amplitudes, respectively.

[f] As calculated using MolProbity (*80*).

Table S2: RMSDs of pairwise structural comparisons between LWY domains of AVRcap1b and PSR2. Provided as a separate file. (**A**) Pairwise comparisons of all LWY domains of *P. infestans* AVRcap1b. (**B**) Pairwise comparisons of all LWY domains of *P. infestans* AVRcap1b to all LWYdomains of *P. sojae* PSR2. Columns and rows highlighted in orange indicate comparisons that involve LWY5 from *P. infestans* AVRcap1b. Provided as a separate file.

Table S3: Shannon's Entropy values for AVRcap1b subclades. Provided as a separate file.

Table S4: Metadata of AVRcap1b and PSR2 orthologs used for the phylogenetic analysis. Provided as a separate file.

Movie S1: Rotating view of the refined AVRcap1b–NbTOL9a ENTH complex fitted to the $2mF_{obs}$–DF_{calc} electron density map. Provided as a separate file.

Movie S2: Rotating view of omit difference maps supporting the placement of AVRcap1b and NbTOL9a ENTH domains. Provided as a separate file.

Data S1: PSI-BLAST output of AVRcap1b search against NCBI non-redundant protein database. Provided as a separate file.

Data S2: Fasta sequences of AVRcap1b and PSR2 orthologs used for the phylogenetic analysis. Provided as a separate file.

Data S3: Fasta sequences of Phytophthora clade 1c AVRcap1b sequences used in this study. Sequences provided are for the mature protein, without including the N-terminal signal peptide or RXLR-DEER motif. Provided as a separate file.

REFERENCES

1. M. P. Contreras, D. Lüdke, H. Pai, A. Toghani, S. Kamoun, NLR receptors in plant immunity: Making sense of the alphabet soup. *EMBO Rep.* **24**, e57495 (2023).

2. B. Sundaram, R. E. Tweedell, S. P. Kumar, T.-D. Kanneganti, The NLR family of innate immune and cell death sensors. *Immunity* **57**, 674–699 (2024).

3. W.-C. Chou, S. Jha, M. W. Linhoff, J. P.-Y. Ting, The NLR gene family: From discovery to present day. *Nat. Rev. Immunol.* **23**, 635–654 (2023).

4. B. P. M. Ngou, P. Ding, J. D. Jones, Thirty years of resistance: Zig-zag through the plant immune system. *Plant Cell* **34**, 1447–1478 (2022).

5. A. Förderer, J. Kourelis, NLR immune receptors: Structure and function in plant disease resistance. *Biochem. Soc. Trans.* **51**, 1473–1483 (2023).

6. L. Derevnina, M. P. Contreras, H. Adachi, J. Upson, A. Vergara Cruces, R. Xie, J. Skłenar, F. L. Menke, S. T. Mugford, D. MacLean, W. Ma, S. A. Hogenhout, A. Goverse, A. Maqbool, C.-H. Wu, S. Kamoun, Plant pathogens convergently evolved to counteract redundant nodes of an NLR immune receptor network. *PLoS Biol.* **19**, e3001136 (2021).

7. M.-Y. Wang, J.-B. Chen, R. Wu, H.-L. Guo, Y. Chen, Z.-J. Li, L.-Y. Wei, C. Liu, S.-F. He, M.-D. Du, The plant immune receptor SNC1 monitors helper NLRs targeted by a bacterial effector. *Cell Host Microbe* **31**, 1792–1803.e7 (2023).

8. C.-h. Wu, L. Derevnina, The battle within: How pathogen effectors suppress NLR-mediated immunity. *Curr. Opin. Plant Biol.* **74**, 102396 (2023).

9. Y. Sugihara, J. Kourelis, M. P. Contreras, H. Pai, A. Harant, M. Selvaraj, A. Toghani, C. Martínez-Anaya, S. Kamoun, Helper NLR immune protein NRC3 evolved to evade inhibition by a cyst nematode virulence effector. *PLOS Genet.* **21**, e1011653 (2025).

10. J. Kourelis, T. Sakai, H. Adachi, S. Kamoun, RefPlantNLR is a comprehensive collection of experimentally validated plant disease resistance proteins from the NLR family. *PLoS Biol.* **19**, e3001124 (2021).

11. H. Adachi, L. Derevnina, S. Kamoun, NLR singletons, pairs, and networks: Evolution, assembly, and regulation of the intracellular immunoreceptor circuitry of plants. *Curr. Opin. Plant Biol.* **50**, 121–131 (2019).

12. J. M. Feehan, B. Castel, A. R. Bentham, J. D. Jones, Plant NLRs get by with a little help from their friends. *Curr. Opin. Plant Biol.* **56**, 99–108 (2020).

13. C.-H. Wu, L. Derevnina, S. Kamoun, Receptor networks underpin plant immunity. *Science* **360**, 1300–1301 (2018).

14. C.-H. Wu, A. Abd-El-Haliem, T. O. Bozkurt, K. Belhaj, R. Terauchi, J. H. Vossen, S. Kamoun, NLR network mediates immunity to diverse plant pathogens. *Proc. Natl. Acad. Sci. U.S.A.* **114**, 8113–8118 (2017).

15. F.-J. Goh, C.-Y. Huang, L. Derevnina, C.-H. Wu, NRC immune receptor networks show diversified hierarchical genetic architecture across plant lineages. *Plant Cell* **36**, 3399–3418 (2024).

16. H. Pai, T. Sakai, A. Posbeyikian, R. Frijters, Y. Sugihara, M. P. Contreras, J. Kourelis, H. Adachi, S. Kamoun, A. Toghani, A hierarchical immune receptor network in lettuce reveals contrasting patterns of evolution in sensor and helper NLRs. bioRxiv 639832 [Preprint] (2025). https://doi.org/10.1101/2025.02.25.639832.

17. M. P. Contreras, H. Pai, M. Selvaraj, A. Toghani, D. M. Lawson, Y. Tumtas, C. Duggan, E. L. H. Yuen, C. E. M. Stevenson, A. Harant, A. Maqbool, C.-H. Wu, T. O. Bozkurt, S. Kamoun, L. Derevnina, Resurrection of plant disease resistance proteins via helper NLR bioengineering. *Sci. Adv.* **9**, eadg3861 (2023).

18. M. P. Contreras, H. Pai, Y. Tumtas, C. Duggan, E. L. H. Yuen, A. V. Cruces, J. Kourelis, H. K. Ahn, K. T. Lee, C. H. Wu, T. O. Bozkurt, L. Derevnina, S. Kamoun, Sensor NLR immune

proteins activate oligomerization of their NRC helpers in response to plant pathogens. *EMBO J.* **42**, e111519 (2023).

19. H. K. Ahn, X. Lin, A. C. Olave-Achury, L. Derevnina, M. P. Contreras, J. Kourelis, C. H. Wu, S. Kamoun, J. D. Jones, Effector-dependent activation and oligomerization of plant NRC class helper NLRs by sensor NLR immune receptors Rpi-amr3 and Rpi-amr1. *EMBO J.* **42**, e111484 (2023).

20. M. P. Contreras, H. Pai, R. Thompson, C. Marchal, J. Claeys, H. Adachi, S. Kamoun, The nucleotide-binding domain of NRC-dependent disease resistance proteins is sufficient to activate downstream helper NLR oligomerization and immune signaling. *New Phytol.* **243**, 345–361 (2024).

21. M. Selvaraj, A. Toghani, H. Pai, Y. Sugihara, J. Kourelis, E. L. H. Yuen, T. Ibrahim, H. Zhao, R. Xie, A. Maqbool, Activation of plant immunity through conversion of a helper NLR homodimer into a resistosome. *PLoS Biol.* **22**, e3002868 (2024).

22. J. Madhuprakash, A. Toghani, M. P. Contreras, A. Posbeyikian, J. Richardson, J. Kourelis, T. O. Bozkurt, M. W. Webster, S. Kamoun, A disease resistance protein triggers oligomerization of its NLR helper into a hexameric resistosome to mediate innate immunity. *Sci. Adv.* **10**, eadr2594 (2024).

23. S. Ma, C. An, A. W. Lawson, Y. Cao, Y. Sun, E. Y. J. Tan, J. Pan, J. Jirschitzka, F. Kümmel, N. Mukhi, Oligomerization-mediated autoinhibition and cofactor binding of a plant NLR. *Nature* **632**, 869–876 (2024).

24. F. Liu, Z. Yang, C. Wang, Z. You, R. Martin, W. Qiao, J. Huang, P. Jacob, J. L. Dangl, J. E. Carette, Activation of the helper NRC4 immune receptor forms a hexameric resistosome. *Cell* **187**, 4877–4889.e15 (2024).

25. V. A. Lopez, B. C. Park, D. Nowak, A. Sreelatha, P. Zembek, J. Fernandez, K. A. Servage, M. Gradowski, J. Hennig, D. R. Tomchick, A bacterial effector mimics a host HSP90 client to undermine immunity. *Cell* **179**, 205–218.e21 (2019).

26. H. S. Karki, S. Abdullah, Y. Chen, D. A. Halterman, Natural genetic diversity in the potato resistance gene RB confers suppression avoidance from *Phytophthora* effector IPI-O4. *Mol. Plant Microbe Interact.* **34**, 1048–1056 (2021).

27. J. Kourelis, M. P. Contreras, A. Harant, H. Pai, D. Lüdke, H. Adachi, L. Derevnina, C.-H. Wu, S. Kamoun, The helper NLR immune protein NRC3 mediates the hypersensitive cell death caused by the cell-surface receptor Cf-4. *PLOS Genet.* **18**, e1010414 (2022).

28. S. Kamoun, O. Furzer, J. D. Jones, H. S. Judelson, G. S. Ali, R. J. Dalio, S. G. Roy, L. Schena, A. Zambounis, F. Panabières, D. Cahill, F. Goverse, The top 10 oomycete pathogens in molecular plant pathology. *Mol. Plant Pathol.* **16**, 413–434 (2015).

29. B. Petre, M. P. Contreras, T. O. Bozkurt, M. H. Schattat, J. Sklenar, S. Schornack, A. Abd-El-Haliem, R. Castells-Graells, R. Lozano-Durán, Y. F. Dagdas, F. L. H. Menke, A. M. E. Jones, J. H. Vossen, S. Robatzek, S. Kamoun, J. Win, Host-interactor screens of *Phytophthora infestans* RXLR proteins reveal vesicle trafficking as a major effector-targeted process. *Plant Cell* **33**, 1447–1471 (2021).

30. J. Win, K. V. Krasileva, S. Kamoun, K. Shirasu, B. J. Staskawicz, M. J. Banfield, Sequence divergent RXLR effectors share a structural fold conserved across plant pathogenic oomycete species. *PLOS Pathog.* **8**, e1002400 (2012).

31. R. H. Jiang, S. Tripathy, F. Govers, B. M. Tyler, RXLR effector reservoir in two *Phytophthora* species is dominated by a single rapidly evolving superfamily with more than 700 members. *Proc. Natl. Acad. Sci. U.S.A.* **105**, 4874–4879 (2008).

32. B. J. Haas, S. Kamoun, M. C. Zody, R. H. Jiang, R. E. Handsaker, L. M. Cano, M. Grabherr, C. D. Kodira, S. Raffaele, T. Torto-Alalibo, Genome sequence and analysis of the Irish potato famine pathogen *Phytophthora infestans*. *Nature* **461**, 393–398 (2009).

33. D. E. Cooke, L. M. Cano, S. Raffaele, R. A. Bain, L. R. Cooke, G. J. Etherington, K. L. Deahl, R. A. Farrer, E. M. Gilroy, E. M. Goss, Genome analyses of an aggressive and invasive lineage of the Irish potato famine pathogen. *PLOS Pathog.* **8**, e1002940 (2012).

34. K. Yoshida, V. J. Schuenemann, L. M. Cano, M. Pais, B. Mishra, R. Sharma, C. Lanz, F. N. Martin, S. Kamoun, J. Krause, The rise and fall of the *Phytophthora infestans* lineage that triggered the Irish potato famine. *eLife* **2**, e00731 (2013).

35. S. Raffaele, R. A. Farrer, L. M. Cano, D. J. Studholme, D. MacLean, M. Thines, R. H. Jiang, M. C. Zody, S. G. Kunjeti, N. M. Donofrio, Genome evolution following host jumps in the Irish potato famine pathogen lineage. *Science* **330**, 1540–1543 (2010).

36. E. K. Zess, Y. F. Dagdas, E. Peers, A. Maqbool, M. J. Banfield, T. O. Bozkurt, S. Kamoun, Regressive evolution of an effector following a host jump in the Irish potato famine pathogen lineage. *PLOS Pathog.* **18**, e1010918 (2022).

37. J. He, W. Ye, D. S. Choi, B. Wu, Y. Zhai, B. Guo, S. Duan, Y. Wang, J. Gan, W. Ma, Structural analysis of *Phytophthora* suppressor of RNA silencing 2 (PSR2) reveals a conserved modular fold contributing to virulence. *Proc. Natl. Acad. Sci. U.S.A.* **116**, 8054–8059 (2019).

38. H. Li, J. Wang, T. Kuan, B. Tang, L. Feng, J. Wang, Z. Cheng, J. Sklenar, P. Derbyshire, M. Hulin, Y. Li, Y. Zhai, Y. Hou, F. L. H. Menke, Y. Wang, W. Ma, Pathogen protein modularity enables elaborate mimicry of a host phosphatase. bioRxiv 539533 [Preprint] (2023). https://doi.org/10.1101/2023.05.05.539533.

39. J. Win, A. Chaparro-Garcia, K. Belhaj, D. Saunders, K. Yoshida, S. Dong, S. Schornack, C. Zipfel, S. Robatzek, S. Hogenhout, in *Cold Spring Harbor Symposia on Quantitative Biology* (Cold Spring Harbor Laboratory Press, 2012), vol. 77, pp. 235–247.

40. A. H. Lovelace, S. Dorhmi, M. T. Hulin, Y. Li, J. W. Mansfield, W. Ma, Effector identification in plant pathogens. *Phytopathology* **113**, 637–650 (2023).

41. J. Wang, H. Li, W. Sun, J. Wang, X. Fang, X. Yang, C. Liu, G. Sheng, W. Ma, Y. Wang, Pathogen effector forms a hexameric phosphatase holoenzyme with host core enzyme to promote disease. bioRxiv [Preprint] 659724 (2025). https://doi.org/10.1101/2025.06.14.659724.

42. J. Moulinier-Anzola, M. Schwihla, L. De-Araújo, C. Artner, L. Jörg, N. Konstantinova, C. Luschnig, B. Korbei, TOLs function as ubiquitin receptors in the early steps of the ESCRT pathway in higher plants. *Mol. Plant* **13**, 717–731 (2020).

43. V. Winter, M.-T. Hauser, Exploring the ESCRTing machinery in eukaryotes. *Trends Plant Sci.* **11**, 115–123 (2006).

44. C. Camacho, G. Coulouris, V. Avagyan, N. Ma, J. Papadopoulos, K. Bealer, T. L. Madden, BLAST+: Architecture and applications. *BMC Bioinformatics* **10**, 1–9 (2009).

45. Z. Abad, T. Burgess, T. Bourret, K. Bensch, S. Cacciola, B. Scanu, R. Mathew, B. Kasiborski, S. Srivastava, K. Kageyama, *Phytophthora*: Taxonomic and phylogenetic revision of the genus. *Stud. Mycol.* **106**, 259–348 (2023).

46. Z. G. Abad, T. I. Burgess, A. J. Redford, J. C. Bienapfl, S. Srivastava, R. Mathew, K. Jennings, *IDphy*: An international online resource for molecular and morphological identification of *Phytophthora*. *Plant Dis.* **107**, 987–998 (2023).

47. J. Abramson, J. Adler, J. Dunger, R. Evans, T. Green, A. Pritzel, O. Ronneberger, L. Willmore, A. J. Ballard, J. Bambrick, Accurate structure prediction of biomolecular interactions with AlphaFold 3. *Nature* **630**, 493–500 (2024).

48. D. M. Prigozhin, K. V. Krasileva, Analysis of intraspecies diversity reveals a subset of highly variable plant immune receptors and predicts their binding sites. *Plant Cell* **33**, 998–1015 (2021).

49. H. Adachi, M. P. Contreras, A. Harant, C.-h. Wu, L. Derevnina, T. Sakai, C. Duggan, E. Moratto, T. O. Bozkurt, A. Maqbool, J. Win, S. Kamoun, An N-terminal motif in NLR immune receptors is functionally conserved across distantly related plant species. *eLife* **8**, e49956 (2019).

50. B. A. Seager, A. Harant, M. P. Contreras, L.-Y. Hou, C.-H. Wu, S. Kamoun, J. Madhuprakash, A plant pathogen effector blocks stepwise assembly of a helper NLR resistosome. *Sci. Adv.* **12**, aeb1931 (2026).

51. Y.-N. Gong, C. Guy, H. Olauson, J. U. Becker, M. Yang, P. Fitzgerald, A. Linkermann, D. R. Green, ESCRT-III acts downstream of MLKL to regulate necroptotic cell death and its consequences. *Cell* **169**, 286–300.e16 (2017).

52. A. J. Jimenez, P. Maiuri, J. Lafaurie-Janvore, S. Divoux, M. Piel, F. Perez, ESCRT machinery is required for plasma membrane repair. *Science* **343**, 1247136 (2014).

53. S. Rühl, K. Shkarina, B. Demarco, R. Heilig, J. C. Santos, P. Broz, ESCRT-dependent membrane repair negatively regulates pyroptosis downstream of GSDMD activation. *Science* **362**, 956–960 (2018).

54. K. Witek, X. Lin, H. S. Karki, F. Jupe, A. I. Witek, B. Steuernagel, R. Stam, C. Van Oosterhout, S. Fairhead, R. Heal, A complex resistance locus in *Solanum americanum* recognizes a conserved *Phytophthora* effector. *Nat. Plants* **7**, 198–208 (2021).

55. E. Weber, C. Engler, R. Gruetzner, S. Werner, S. Marillonnet, A modular cloning system for standardized assembly of multigene constructs. *PLOS ONE* **6**, e16765 (2011).

56. C. Engler, M. Youles, R. Gruetzner, T.-M. Ehnert, S. Werner, J. D. Jones, N. J. Patron, S. Marillonnet, A golden gate modular cloning toolbox for plants. *ACS Synth. Biol.* **3**, 839–843 (2014).

57. J. Wen, T. Arakawa, J. S. Philo, Size-exclusion chromatography with on-line light-scattering, absorbance, and refractive index detectors for studying proteins and their interactions. *Anal. Biochem.* **240**, 155–166 (1996).

58. G. Winter, xia2: An expert system for macromolecular crystallography data reduction. *J. Appl. Cryst.* **43**, 186–190 (2010).

59. M. Winn, An overview of the CCP4 project in protein crystallography: An example of a collaborative project. *J. Synchrotron Radiat.* **10**, 23–25 (2003).

60. R. Evans, M. O'Neill, A. Pritzel, N. Antropova, A. Senior, T. Green, A. Žídek, R. Bates, S. Blackwell, J. Yim, Protein complex prediction with AlphaFold-Multimer. bioRxiv 463034 [Preprint] (2021). https://doi.org/10.1101/2021.10.04.463034.

61. M. Mirdita, K. Schütze, Y. Moriwaki, L. Heo, S. Ovchinnikov, M. Steinegger, ColabFold: Making protein folding accessible to all. *Nat. Methods* **19**, 679–682 (2022).

62. D. Liebschner, P. V. Afonine, M. L. Baker, G. Bunkóczi, V. B. Chen, T. I. Croll, B. Hintze, L.-W. Hung, S. Jain, A. J. McCoy, Macromolecular structure determination using X-rays, neutrons and electrons: Recent developments in *Phenix*. *Acta Crystallogr. D. Struct. Biol.* **75**, 861–877 (2019).

63. A. J. McCoy, R. W. Grosse-Kunstleve, P. D. Adams, M. D. Winn, L. C. Storoni, R. J. Read, Phaser crystallographic software. *J. Appl. Cryst.* **40**, 658–674 (2007).

64. P. Emsley, B. Lohkamp, W. G. Scott, K. Cowtan, Features and development of Coot. *Acta Crystallogr. D Biol. Crystallogr.* **66**, 486–501 (2010).

65. G. N. Murshudov, P. Skubák, A. A. Lebedev, N. S. Pannu, R. A. Steiner, R. A. Nicholls, M. D. Winn, F. Long, A. A. Vagin, REFMAC5 for the refinement of macromolecular crystal structures. *Acta Crystallogr. D Biol. Crystallogr.* **67**, 355–367 (2011).

66. R. A. Nicholls, M. Fischer, S. McNicholas, G. N. Murshudov, Conformation-independent structural comparison of macromolecules with ProSMART. *Acta Crystallogr. D Biol. Crystallogr.* **70**, 2487–2499 (2014).

67. E. F. Pettersen, T. D. Goddard, C. C. Huang, E. C. Meng, G. S. Couch, T. I. Croll, J. H. Morris, T. E. Ferrin, UCSF ChimeraX: Structure visualization for researchers, educators, and developers. *Protein Sci.* **30**, 70–82 (2021).

68. A. Toghani, S. Kamoun, M. P. Contreras, Supplementary material for "An effector from the potato late blight pathogen bridges ENTH-domain protein TOL9a to an activated helper NLR to suppress immunity," Zenodo (2025); doi.org/10.5281/zenodo.15600152.

69. L. Fu, B. Niu, Z. Zhu, S. Wu, W. Li, CD-HIT: Accelerated for clustering the next-generation sequencing data. *Bioinformatics* **28**, 3150–3152 (2012).

70. K. Katoh, D. M. Standley, MAFFT multiple sequence alignment software version 7: Improvements in performance and usability. *Mol. Biol. Evol.* **30**, 772–780 (2013).

71. J. L. Steenwyk, T. J. Buida III, Y. Li, X.-X. Shen, A. Rokas, ClipKIT: A multiple sequence alignment trimming software for accurate phylogenomic inference. *PLoS Biol.* **18**, e3001007 (2020).

72. J. Hausser, K. Strimmer, Entropy inference and the James-Stein estimator, with application to nonlinear gene association networks. *J. Mach. Learn. Res.* **10**, 1469–1484 (2009).

73. O. Wagih, ggseqlogo: A versatile R package for drawing sequence logos. *Bioinformatics* **33**, 3645–3647 (2017).

74. H. Wickham, "Getting Started with ggplot2," in *ggplot2: Elegant Graphics for Data Analysis* (Springer, 2016), pp. 11–31.

75. D. H. Huson, C. Scornavacca, Dendroscope 3: An interactive tool for rooted phylogenetic trees and networks. *Syst. Biol.* **61**, 1061–1067 (2012).

76. M. N. Price, P. S. Dehal, A. P. Arkin, FastTree 2–approximately maximum-likelihood trees for large alignments. *PLOS ONE* **5**, e9490 (2010).

77. M. E. Segretin, M. Pais, M. Franceschetti, A. Chaparro-Garcia, J. I. Bos, M. J. Banfield, S. Kamoun, Single amino acid mutations in the potato immune receptor R3a expand response to *Phytophthora* effectors. *Mol. Plant Microbe Interact.* **27**, 624–637 (2014).

78. D. MacLean, TeamMacLean/besthr: Initial Release, Zenodo (2019); https://zenodo.org/records/3374507.

79. A. Toghani, amiralito/AVRcap1b: Repository Release, versio 1.0.0, Zenodo (2026); https://doi.org/10.5281/zenodo.19554461.

80. I. W. Davis, A. Leaver-Fay, V. B. Chen, J. N. Block, G. J. Kapral, X. Wang, L. W. Murray, W. B. Arendall III, J. Snoeyink, J. S. Richardson, MolProbity: All-atom contacts and structure validation for proteins and nucleic acids. *Nucleic Acids Res.* **35**, W375–W383 (2007).
